# Academic Self-Pressure and Physiological Responses in Adolescents: A Pilot Experimental Study on the Moderating Role of an Escape Room-Based Physical Activity Intervention on Cognitive and Academic Outcomes

**DOI:** 10.3390/ijerph22060948

**Published:** 2025-06-17

**Authors:** Francesca Latino, Domenico Tafuri, Francesco Tafuri

**Affiliations:** 1 Department of Human, Educational and Sport Sciences, Pegaso University, 80143 Napoli, Italy; 2 Department of Medical, Motor and Wellness Sciences, University of Naples “Parthenope”, 80133 Napoli, Italy; domenico.tafuri@uniparthenope.it; 3 Heracle Lab Research in Educational Neuroscience, Niccolò Cusano University, 00166 Roma, Italy; francesco.tafuri@unicusano.it

**Keywords:** heart rate variability, stress, cognitive performance, physical activity, movement-based learning

## Abstract

Academic self-pressure is a significant source of stress for students, with physiological and cognitive implications that can influence academic performance. This study investigated the impact of academic self-pressure on heart rate variability (HRV) and cognitive performance, exploring the moderating role of physical activity through an experimental intervention. A randomized controlled trial (RCT) was conducted on a sample of 50 secondary school students, divided into an experimental group and a control group. The intervention, lasting 16 weeks, integrated physical activity based on escape room challenges with the traditional school curriculum. The results show that the experimental group recorded a significant improvement in HRV, a reduction in perceived stress, and an increase in cognitive performance, working memory, and academic achievement. Correlational and regression analyses highlighted the key role of physiological stress regulation in academic success. The findings emphasize the potential of integrating movement-based learning activities, such as escape room interventions, into school curricula as an effective strategy to enhance students’ stress regulation, executive functioning, and academic performance. By improving physiological self-regulation and cognitive efficiency, this approach supports a more holistic educational model that addresses both academic outcomes and student well-being. These results suggest that incorporating physically active, cognitively engaging tasks into the classroom may foster resilience, motivation, and adaptive coping skills, offering practical value for educational policy and classroom practice.

## 1. Introduction

Academic self-pressure refers to the internalized stress that students place upon themselves to achieve high academic standards. Unlike external academic pressure, which stems from teachers, parents, or institutional expectations, self-imposed pressure originates from personal aspirations, perfectionism, and the desire to excel [1]. While a certain level of self-motivation can drive academic success, excessive academic self-pressure can lead to chronic stress, anxiety, and burnout [2]. In modern educational settings, where competition is high and expectations continue to rise, understanding the physiological and psychological effects of academic self-pressure are crucial [3,4]. When students experience academic self-pressure, their bodies engage in a physiological stress response primarily regulated by the autonomic nervous system (ANS) and the hypothalamic–pituitary–adrenal (HPA) axis [5]. This response involves the release of stress hormones such as cortisol and adrenaline, which prepare the body for immediate challenges but can become detrimental if prolonged [6]. One key physiological indicator of stress is heart rate variability (HRV), a measure of autonomic nervous system regulation that reflects the balance between sympathetic (fight-or-flight) and parasympathetic (rest-and-digest) activity [7]. Reduced HRV has been associated with chronic stress, anxiety, and cognitive fatigue, making it a valuable marker for assessing the physiological impact of academic self-pressure [8]. HRV is a crucial biomarker in understanding the interaction between physiological stress and cognitive performance. High HRV indicates a well-regulated autonomic nervous system, allowing for better stress adaptation, emotional regulation, and cognitive flexibility [9]. Conversely, low HRV has been linked to impaired executive function, reduced working memory capacity, and increased susceptibility to stress-related disorders [10]. Given that academic self-pressure often leads to chronic stress and decreased HRV, understanding how interventions such as physical activity influence HRV can provide valuable insights into supporting student well-being and academic achievement.

Exercise has long been recognized as a powerful tool for enhancing both physical and mental health. Regular physical activity is associated with improvements in mood, cognitive function, and stress resilience [11]. From a physiological standpoint, the positive effects of exercise on mental health are mediated by several neurobiological mechanisms. Physical activity stimulates the release of Brain-Derived Neurotrophic Factor (BDNF), which supports synaptic plasticity, neurogenesis, and overall brain resilience, particularly in regions involved in learning and emotion regulation, such as the hippocampus and prefrontal cortex [12]. Additionally, regular aerobic exercise has been shown to reduce basal cortisol levels and modulate HPA axis reactivity, thereby decreasing the chronic overactivation often observed in individuals under persistent stress [13]. This downregulation of stress hormones contributes to emotional balance and cognitive stability. Furthermore, exercise activates the vagus nerve, enhancing parasympathetic tone and increasing HRV, which in turn promotes executive functioning, attentional control, and emotional regulation [14]. These physiological pathways suggest that physical activity not only counters the detrimental effects of academic self-pressure but also creates neurobiological conditions favorable to learning and psychological well-being [15]. Aerobic exercise in particular has demonstrated significant benefits in mitigating the negative effects of stress on HRV and cognitive performance [16]. By incorporating physical activity into daily routines, students may be able to counteract the physiological consequences of academic self-pressure and enhance their academic outcomes. The relationship between exercise, HRV, and academic performance is supported by a growing body of research. Studies have shown that students who engage in regular physical activity exhibit improved concentration, memory retention, and problem-solving abilities [17,18]. Additionally, physical activity has been linked to better emotional regulation and resilience, which are essential for coping with academic stress [19]. Chang et al. [20], for instance, found that acute bouts of exercise can significantly improve executive function in young populations. Similarly, Khan and Hillman [21] demonstrated that regular aerobic training enhances attention and academic achievement. However, most of these studies relied on traditional physical activity protocols, without integrating academic content or active learning strategies. Recent literature has also examined classroom-based movement interventions (e.g., active breaks or standing lessons) and their effects on attention and well-being, yet few have combined structured aerobic movement with curriculum-driven cognitive challenges [22,23,24].

Given that HRV serves as a physiological bridge between stress and cognitive performance, investigating how exercise influences HRV in students experiencing academic self-pressure can provide valuable insights into effective stress-management strategies.

Among emerging movement-based learning strategies, the use of educational escape rooms has gained increasing attention in recent years [25]. An escape room is a gamified, time-bound activity in which participants solve puzzles and challenges in a cooperative setting to achieve specific educational objectives. In the school context, this format merges cognitive engagement with moderate physical activity, fostering collaboration, critical thinking, and problem-solving skills [26]. In Italy, several pilot projects have successfully implemented escape room interventions in secondary schools, often within interdisciplinary curricula. For example, the “Scuola in Gioco” initiative in Emilia-Romagna and the “Classe in Fuga” project in Lombardy integrated physical movement, logic challenges, and curricular content to boost student motivation and engagement. Early evidence from these experiences has shown positive outcomes in terms of student participation, self-efficacy, and learning retention [27]. Internationally, similar interventions have reported improvements in teamwork, cognitive flexibility, and emotional regulation among adolescents [28]. However, most existing implementations have lacked a rigorous physiological evaluation, particularly concerning stress biomarkers such as HRV.

Indeed, it can be said that despite the growing body of research investigating the impact of academic stress on students’ physiological and cognitive functioning, a significant gap remains in understanding how structured physical activity interventions can buffer these effects, particularly within real-world educational settings [29,30,31]. Most prior studies have adopted correlational or observational designs, offering limited insight into causal relationships. Furthermore, while the positive influence of exercise on stress regulation and cognitive performance has been widely acknowledged, few studies have examined the integration of physical activity into curriculum-based, cognitively engaging formats such as escape rooms [32]. These active learning environments combine physical movement, problem-solving, and academic content, yet their potential to regulate physiological stress (e.g., HRV) and enhance academic outcomes has not been thoroughly investigated in adolescents. Therefore, this study addresses this gap by implementing a randomized controlled trial to examine whether a gamified physical activity intervention can moderate the effects of academic self-pressure on students’ stress physiology and learning performance. Moreover, the present study differentiates itself from previous investigations by incorporating HRV as a physiological index of intervention efficacy, thus bridging a crucial gap between educational innovation and psychophysiological evaluation.

In fact, the rationale for conducting this study stems from the increasing prevalence of academic-related stress in adolescents and the limited availability of interventions that address both its psychological and physiological dimensions. While educational systems continue to prioritize academic outcomes, there is a growing recognition of the need to support students’ mental health and emotional resilience [33]. Academic self-pressure in particular represents a unique form of stress that is often overlooked in school-based interventions. Given its association with impaired HRV and executive dysfunction, there is a pressing need for evidence-based strategies capable of enhancing stress regulation while also promoting academic success. By integrating movement into cognitively stimulating activities, the escape room approach aligns with contemporary models of embodied cognition, which emphasize the role of physical engagement in knowledge acquisition and emotional balance [34]. This study therefore seeks to validate a multidimensional intervention that responds to both the academic and psychophysiological needs of adolescent learners.

Stemming from this background, the present study proposes the following hypotheses: (1) Students in the experimental group show significant improvements in HRV, perceived stress, academic self-pressure, well-being, and cognitive performance (working memory and cognitive flexibility) compared to the control group; (2) HRV emerges as a significant predictor of academic performance and cognitive outcomes, supporting its role as a physiological mediator between stress regulation and learning; (3) The integration of a curriculum-based, physically active escape room intervention produces measurable benefits in both psychological and academic domains, offering a novel, scalable approach for reducing the negative effects of academic self-pressure in adolescent students.

## 2. Materials and Methods

### 2.1. Study Design

This pilot study employed a randomized controlled trial (RCT) design to investigate the impact of an escape room-based physical activity intervention on academic self-pressure, heart rate variability (HRV), cognitive performance, well-being, and academic achievement. The study lasted for 16 weeks, during which intervention was conducted three times per week, with each session lasting 50 min. Participants were randomly assigned to either an intervention group, which engaged in the escape room-based physical activity program integrated with curricular lessons, or a control group that followed the standard curriculum without additional physical activity interventions. The randomization followed a class-based allocation to avoid disrupting the regular curricular schedule of students. The study was conducted from September 2024 to December 2024 and complied with the ethical guidelines outlined in the Declaration of Helsinki and its subsequent revisions. The study was approved by the Department of Medical, Motor, and Wellness Sciences at the University of Naples “Parthenope” (DiSMMeB Prot. N. 88592/2024). Informed consent was obtained from both students and their legal guardians before participation.

### 2.2. Participants

Participants in this study were recruited from two second-year classes of a public secondary school located in a mid-sized town in southern Italy. The school serves a mixed socioeconomic population and follows a general academic curriculum with a focus on humanities and language studies. The area is characterized by moderate urban development, with limited access to extracurricular physical activity programs, particularly structured sport or wellness services within the school. The school had no ongoing health or mindfulness initiatives at the time of the study, ensuring that no overlapping interventions could confound the results.

The final sample consisted of 50 students (N = 50), with 23 males (46%) and 27 females (54%), all between 14 and 16 years old (M = 15.2, SD = 0.6). All students from the selected classes were invited to participate, and all met the inclusion criteria, which required (a) regular school attendance (≥90% in the previous term), (b) absence of medical conditions interfering with physical activity or HRV measurement, and (c) willingness to participate in all phases of the study (Table 1).

Exclusion criteria included (a) diagnosed cardiovascular conditions (e.g., arrhythmia, hypertension), (b) neurological or developmental disorders (e.g., epilepsy, ADHD), (c) chronic illness requiring medications that influence autonomic regulation (e.g., antidepressants, beta-blockers), and (d) regular participation in competitive sports. These criteria aimed to control for baseline variability in physiological stress responses and physical conditioning.

Demographically, most students belonged to middle-income households. No participant reported current or past psychological diagnoses or formal engagement in structured mindfulness, cognitive behavioral therapy, or other stress reduction programs. According to responses from an initial health and lifestyle questionnaire, 80% of students reported engaging in light to moderate physical activity (e.g., walking, recreational sports) once or twice per week, while the remaining 20% described themselves as predominantly sedentary. This distribution was comparable across groups.

To explore psychological and cognitive comparability, all participants completed a brief baseline screening battery assessing academic self-efficacy, perceived stress, and engagement with schoolwork. No statistically significant differences were found between the experimental and control groups on these measures (*p* > 0.05), confirming group homogeneity prior to the intervention.

An a priori power analysis using G*Power 3.1 determined that a sample size of 50 is sufficient to detect medium effect sizes (d = 0.5) with 80% power at a 5% significance level. This sample size was also consistent with the logistical constraints of the school environment and available instructional hours.

To minimize contamination between conditions and preserve curricular structure, class-level randomization was used: one class was assigned to the experimental group (n = 25) and the other to the control group (n = 25). Both classes were taught by the same Italian language teacher, ensuring instructional consistency, and shared the same schedule for academic activities. The intervention was conducted during regular school hours, with no interference from external programs or schedule changes, thereby minimizing the influence of confounding environmental factors.

### 2.3. Procedures

The study was implemented over a total duration of 16 weeks, during which participants were assessed and monitored according to a predefined schedule. All procedures followed a structured protocol designed to ensure consistency in intervention delivery, timing of measurements, and data collection across both groups.

Prior to the start of the intervention (Week 0), baseline assessments were conducted for all participants, including physiological recordings (HR and HRV), cognitive tasks, psychological questionnaires, and academic data collection. Students were familiarized with the instruments and procedures through a preliminary orientation session to minimize novelty effects and improve compliance.

The experimental group began the intervention in Week 1, attending physical activity sessions integrated within regular class hours. To ensure uniformity, all sessions were conducted using standardized lesson plans, and attendance was tracked systematically using individual logs. A designated observer from the research team attended sessions to monitor protocol adherence, provide support, and collect qualitative notes on student engagement and group dynamics.

At Week 16, post-intervention data were collected following the same procedures and order as the baseline, maintaining time-of-day consistency to control for circadian effects on HRV and cognitive performance. HRV was evaluated utilizing the Polar H10 chest strap and data were processed using Kubios HRV software (v 4.1.2.1). Data from HR monitors were downloaded and processed using the same protocol across the two time points to ensure data integrity.

The control group was assessed on the same schedule as the experimental group but did not receive any additional activities. Teachers were instructed to avoid introducing novel teaching methods or extracurricular tasks during the study period, ensuring a stable curricular environment. To reduce potential bias, data collectors were blinded to group allocation during assessments and data entry.

Weekly team meetings were held throughout the intervention to review implementation fidelity, address unforeseen logistical issues, and ensure ethical and methodological compliance. All deviations from protocol (e.g., absences, technical issues) were documented in a centralized research log.

### 2.4. Intervention Program

The intervention program spanned 16 weeks and included 48 sessions (3 sessions/week, 50 min each). It was structured into four progressive phases, each lasting four weeks, gradually increasing in both physical and cognitive complexity. The activities were designed to integrate physical exertion with curriculum content in Italian language and literature through escape room-inspired challenges.

Each session included a standardized warm-up (5–7 min), core activity (35–40 min), and cool-down (5 min). The physical component progressed from light aerobic movements (e.g., dynamic stretching, balance games) to moderate and eventually high-intensity drills (e.g., HIIT circuits, agility and reaction tasks).

The cognitive tasks followed a pedagogical scaffold aligned with learning goals: from basic grammar and vocabulary exercises to textual interpretation and thematic literary debates. All activities were designed to promote teamwork, decision-making, problem-solving, and movement-based encoding of academic content.

Teachers and trained sports educators co-conducted the sessions to ensure both academic relevance and physical safety. Attendance and participation were logged throughout the program.

**Phase 1: Foundation (Weeks 1–4)**
Focus: Basic linguistic and physical skills

- Physical Activity: Light aerobic exercises (jumping jacks, dynamic stretching, balance exercises).
- Escape Room Challenges:
  ○ Grammar and Vocabulary Games:
    ■ Word association relays: Students jog in place while linking words in semantic categories.
    ■ Sentence-building obstacle course: Teams navigate a course collecting words to form grammatically correct sentences.
  ○ Basic Riddles: Deciphering simple language-based clues to unlock next stages.
  ○ Collaboration Tasks: Team-based warm-ups to foster group dynamics.

**Phase 2: Textual Analysis in Motion (Weeks 5–8)**
Focus: Reading comprehension and information extraction

- Physical Activity: Moderate-intensity exercises (obstacle courses, relay races, agility drills).
- Escape Room Challenges:
  ○ Text-Based Missions:
    ■ Extracting key information from short texts while completing a movement task (e.g., scanning a passage while balancing on a beam).
    ■ “Find the Clue” Race: Students sprint to retrieve hidden excerpts, then arrange them in logical sequence.
  ○ Team Strategy Tasks: Groups compete to summarize information efficiently under time pressure.
  ○ Puzzle Locks: Matching key words or main ideas to corresponding padlocks to progress in the escape room.

**Phase 3: Literary Comprehension and Coordination (Weeks 9–12)**
Focus: Advanced literary interpretation and movement-based cognition

- Physical Activity: Increased complexity in coordination drills, partner exercises, and endurance-based challenges.
- Escape Room Challenges:
  ○ Summarizing While Moving:
    ■ Teams perform a coordination exercise (e.g., dribbling or balancing) while summarizing literary passages.
  ○ Symbolic Puzzle Challenge:
    ■ Identifying metaphors, symbols, and themes in texts and matching them to corresponding objects within the escape room.
  ○ Memory and Recall Relays:
    ■ Students retrieve parts of a poem or prose and reconstruct it collaboratively while performing movement-based tasks.

**Phase 4: High-Intensity Training and Thematic Analysis (Weeks 13–16)**
Focus: Advanced critical thinking, problem-solving, and teamwork

- Physical Activity: High-intensity interval training (HIIT), reaction drills, and cooperative strength challenges.
- Escape Room Challenges:
  ○ Timed Literary Debates and Physical Strategy Games:
    ■ Teams engage in analytical discussions about literary themes while solving movement-based challenges.
  ○ Problem-Solving Under Pressure:
    ■ Combining physical endurance with linguistic challenges such as unlocking a final puzzle using analyzed literary elements.
  ○ Grand Escape Challenge:
    ■ A final multi-layered challenge integrating all skills developed, requiring teamwork, physical agility, and deep textual analysis.

By combining physical movement with linguistic and literary learning, this program aims to create a more engaging and dynamic educational experience. Through interactive problem-solving activities, students are encouraged to deepen their reading comprehension and textual analysis skills in a stimulating and unconventional way. The collaborative nature of the escape room tasks fosters teamwork and communication, requiring participants to strategize and work together to overcome challenges.

Additionally, the integration of progressive physical exercises supports both physical well-being and cognitive endurance, helping students stay focused and energized throughout the learning process. By incorporating game-based and experiential methods, the program also seeks to enhance student motivation, making language learning more enjoyable and immersive.

### 2.5. Measures

To comprehensively assess the impact of the intervention, multiple measures were employed to evaluate physiological stress responses, self-imposed academic pressure, cognitive performance, and overall well-being. Data were collected at three key moments: before the intervention (baseline) and after its conclusion (post-intervention). This approach allowed for a detailed analysis of how students’ physiological and psychological responses evolved over time.

All psychological scales and cognitive tests were administered in paper-and-pencil format by members of the research team who were present in the classroom to provide standardized instructions, ensure procedural consistency, and answer any clarification questions. The administration took place in group settings, with students seated at individual desks to ensure privacy and minimize distractions. All measures were collected during regular school hours, in the students’ usual classroom environment, with sessions conducted in a quiet and controlled setting. Each assessment session lasted approximately 45–60 min, depending on the number of instruments administered. The full battery of tests, including self-report questionnaires (AESI, PSS-10, Well-Being Scale), the Digit Span Test, the Stroop Task, and the demographic/health questionnaire, was administered on the same day to each group at two time points: baseline (2 September 2024) and post-intervention (23 December 2024). To reduce fatigue and ensure attention, short breaks (2–3 min) were included between test sections when needed. The same procedures were followed for both experimental and control groups, and all assessments were scheduled at the same time of day (mid-morning) to control for circadian variations in physiological and cognitive functioning.

#### 2.5.1. Heart Rate (HR) and Heart Rate Variability (HRV)

Heart rate (HR) and heart rate variability (HRV) were key physiological measures employed in this study to assess autonomic nervous system (ANS) activity and stress regulation. HRV in particular is recognized as a fundamental quantitative indicator of the fluctuations in time between consecutive heartbeats, commonly referred to as R-R intervals [35]. This metric is widely considered a reliable biomarker of physiological and psychological adaptability, as it reflects the dynamic balance between sympathetic and parasympathetic nervous system activity [36].

A higher HRV is indicative of a well-regulated autonomic system capable of efficiently adapting to external stressors, which is associated with better overall health, improved cognitive function, and enhanced executive processing abilities. Conversely, lower HRV suggests compromised ANS responsiveness, often linked to elevated stress levels, cognitive fatigue, and physical overexertion, making it a critical parameter for evaluating physiological resilience [37].

To ensure accurate and practical HRV monitoring, this study utilized the Polar H10 (Polar Electro–Finnish) chest strap, which was selected for its high reliability and field applicability [38]. HRV data were processed using Kubios HRV software (Kubios HRV Scientific v 4.1.2.1—Kubios Oy, Finnish, Kuopio, Finland). All recordings were conducted in a quiet room with participants in a seated position to ensure consistency. Traditional electrocardiogram (ECG) devices, while highly precise, may be cumbersome in dynamic, real-world environments; thus, the Polar H10 presents a viable alternative for HRV assessment in both resting and active conditions [39]. Research conducted by Gilgen-Ammann et al. [40] has validated the accuracy of the Polar H10 in comparison with a three-lead ECG Holter monitor, demonstrating an average discrepancy of just 0.23 ± 26.8 ms in R-R interval measurements. The device exhibited exceptional signal quality (99.6%) and maintained a strong correlation (r = 0.997) with the ECG monitor, with no statistically significant differences between the two methods (*p* = 0.208).

Additionally, the quality of the R-R interval signal was evaluated by analyzing missing data points and detection errors. Under low to moderate physical exertion, the Polar H10 showed performance equivalent to that of the Holter monitor. However, during high-intensity activity, the Polar H10 outperformed the Holter, recording only 74 errors (99.4% signal accuracy) in the R-R intervals, whereas the Holter monitor registered 1332 errors (89.9% signal accuracy). These findings reinforce the suitability of the Polar H10 as a reliable tool for HRV analysis in athletic and educational settings, particularly when investigating the relationship between physiological stress regulation, cognitive performance, and academic self-pressure.

#### 2.5.2. Academic Self-Pressure (AESI–Expectations of Self Subscale)

The extent to which students imposed high academic expectations on themselves was assessed using the Expectations of Self subscale from the Academic Expectations Stress Inventory (AESI) [41]. This four-item scale asked students to rate their experiences on a five-point Likert scale, ranging from 1 (“Strongly disagree”) to 5 (“Strongly agree”), where higher scores indicated greater pressure. Previous research has shown that this scale is highly reliable and correlates with perfectionism and academic stress. The AESI was translated into Italian following the forward–backward translation method to ensure semantic and conceptual equivalence. A pilot test was conducted with a sample of 20 students not included in the main study to assess item clarity and cultural appropriateness. Cronbach’s alpha for the subscale in our sample was 0.82, indicating good internal consistency. Example items included:

- *“I feel stressed when I do not meet my own academic expectations.”*
- *“I push myself very hard to succeed academically.”*

#### 2.5.3. Perceived Stress Scale (PSS-10)

Perceived stress was measured using the Italian validated version of the PSS-10 [42], a 10-item self-report tool assessing the degree to which individuals perceive situations in their lives as stressful. Items were scored on a 5-point Likert scale (0 = never, 4 = very often), with reverse scoring applied where appropriate. Cronbach’s alpha in our sample was 0.87. This widely used instrument evaluates how unpredictable, uncontrollable, and overwhelming individuals perceive their lives to be over the past month.

The PSS-10 has been widely used in adolescent populations and shows strong concurrent validity with biomarkers such as cortisol, and good construct validity in the context of academic stress research. Example items included:

- *“In the last month, how often have you felt nervous and stressed?”*
- *“In the last month, how often have you felt that things were going your way?” (reverse-scored).*

#### 2.5.4. Well-Being Questionnaire

Subjective well-being was assessed using a custom short form derived from Ryff’s Psychological Well-Being (PWB) scale [43]. The adapted version included 6 items covering academic satisfaction, emotional balance, and school-related quality of life. Responses were recorded on a 7-point Likert scale (1 = strongly disagree, 7 = strongly agree). The adapted items were reviewed by a panel of experts in educational psychology and piloted with students for face validity. Cronbach’s alpha was 0.84, supporting internal reliability. The scale was positively correlated with academic engagement and inversely correlated with perceived stress, confirming its convergent validity. Example items included:

- *“I feel satisfied with my academic experience.”*
- *“I feel emotionally balanced in my daily school life.”*

Pilot studies have demonstrated that this scale has strong internal consistency and is positively associated with life satisfaction while negatively correlated with stress and anxiety.

#### 2.5.5. Working Memory and Executive Function (Digit Span Test and Stroop Task)

Cognitive performance was evaluated through a combination of working memory and executive function tests. The Digit Span Test [44] was used to assess working memory capacity, requiring students to recall sequences of numbers in both forward and backward order. This test provided insights into students’ ability to retain and manipulate information, which is crucial for learning and academic performance. The Digit Span Subtest has demonstrated strong internal consistency in adolescents, with reported Cronbach’s alpha values ranging from 0.84 to 0.88 depending on age group and task version (forward or backward).

The Stroop Task [45], a well-known cognitive flexibility test, measured students’ ability to manage attentional control and inhibit automatic responses. By requiring participants to name the ink color of incongruent words (e.g., the word “blue” written in red ink), this test evaluated cognitive flexibility, processing speed, and selective attention, all essential for problem-solving and academic success. Although the Stroop Task is a performance-based cognitive test rather than a questionnaire, prior studies have reported acceptable internal consistency for reaction time-based interference scores, with Cronbach’s alpha estimates between 0.75 and 0.82 and test–retest reliability coefficients as high as 0.91.

#### 2.5.6. Reading Comprehension and Analytical Skills

To measure students’ ability to analyze and interpret texts, standardized textual analysis tasks were used. The reading comprehension tasks were scored using a rubric-based assessment aligned with national education standards. In a pilot study conducted on a separate sample (N = 20), the scale demonstrated acceptable internal reliability, with a Cronbach’s alpha of 0.78 [46]. These tasks required students to engage in the following activities:

- Extract key information from texts;
- Summarize content concisely;
- Analyze literary themes within structured assessments.

By integrating these assessments into the intervention, researchers could evaluate whether the escape room-based physical activity program contributed to improved linguistic and critical thinking skills.

#### 2.5.7. Academic Performance (Grades in Italian Language and Literature)

Students’ academic performance was tracked through their grades in Italian language and literature, both in written assignments and oral assessments. These grades served as an objective indicator of students’ progress in mastering reading, writing, and analytical skills over the course of the program.

### 2.6. Statistical Analysis

To evaluate the effectiveness of the intervention across physiological, psychological, cognitive, and academic domains, a comprehensive set of statistical analyses was conducted using IBM SPSS Statistics version 25.0 (IBM Corp., Armonk, NY, USA), with statistical significance set at *p* < 0.05 (two-tailed). Prior to the main analyses, all variables were tested for normality using the Shapiro–Wilk test and visual inspection of histograms and Q-Q plots, while Levene’s test was used to assess homogeneity of variances. Within-group comparisons (pre- vs. post-intervention) were analyzed using paired-sample *t*-tests for normally distributed variables and Wilcoxon signed-rank tests for non-normal or ordinal data to examine changes in heart rate (HR), heart rate variability (HRV), perceived stress (PSS-10), academic self-pressure (AESI), well-being, cognitive performance (Digit Span, Stroop Task), reading comprehension, and academic grades. Between-group differences were assessed using independent-sample *t*-tests or Mann–Whitney U tests on delta scores (Δ = post − pre) for each variable. To investigate the interaction effects of group (experimental vs. control) and time (pre vs. post), repeated-measures ANOVAs were conducted, with Greenhouse–Geisser corrections applied where sphericity assumptions were violated; for non-parametric data, Friedman tests were used as alternatives. Post hoc comparisons were corrected using the Bonferroni method to control for type I error. Correlation analyses, using Pearson’s r for normally distributed variables and Spearman’s rho for ordinal or non-normally distributed data, were performed to explore the associations between HRV changes and other outcome measures such as perceived stress, academic self-pressure, well-being, executive function, working memory, and academic performance. Additionally, multiple linear regression models were employed to examine whether changes in HRV predicted improvements in cognitive performance and academic outcomes, with models controlling for baseline scores, age, sex, and physical activity level where appropriate. Model assumptions and multicollinearity were tested to ensure robustness. Effect sizes were reported to complement statistical significance testing, using Cohen’s d for *t*-tests, partial eta squared (η^2^) for ANOVAs, and r for non-parametric tests, providing a clearer interpretation of the magnitude of observed effects.

## 3. Results

### 3.1. Confirmatory Factor Analysis and Construct Validity of the Measurement Scales

To assess the construct validity of the instruments used, separate Confirmatory Factor Analyses (CFAs) were conducted for each scale: the Academic Self-Pressure Inventory (AESI), the Perceived Stress Scale (PSS-10), and the Well-Being Scale. Each scale was modeled as a unidimensional construct, in line with the existing literature. Model fit was evaluated based on commonly accepted thresholds: Comparative Fit Index (CFI) ≥ 0.90, Tucker–Lewis Index (TLI) ≥ 0.90, Root Mean Square Error of Approximation (RMSEA) ≤ 0.08, and Standardized Root Mean Square Residual (SRMR) ≤ 0.08. The results of the CFA are presented in Table 2.

All CFA models demonstrated a good fit to the data, with CFI and TLI values above 0.90 and RMSEA and SRMR values below 0.08. These findings support the construct validity of the three scales in the sample analyzed.

### 3.2. Paired t-Test/Wilcoxon Test Results (Pre–Post for Each Group)

To assess the effectiveness of physical activity on students’ physiological, psychological, and academic outcomes, a paired *t*-test (or Wilcoxon test when normality assumptions were not met) was conducted. This test was chosen because it allows for a comparison of pre- and post-intervention measurements within the same group of participants, determining whether the observed differences are statistically significant (Table 3).

Within-group analyses revealed significant pre–post improvements in the experimental group across several key measures, including HRV, perceived stress, academic self-pressure, well-being, cognitive flexibility (Stroop Task), and working memory (Digit Span). These changes suggest that the escape room-based intervention, which integrated moderate physical activity with cognitively demanding academic tasks, effectively enhanced students’ physiological and psychological self-regulation. The increase in HRV indicates improved autonomic balance, often associated with better emotional regulation and stress resilience. Simultaneously, the observed reductions in perceived stress and academic self-pressure may reflect not only a physiological benefit but also a cognitive reframing of academic challenges as more manageable, possibly due to the mastery experiences embedded in the gamified format. In contrast, the control group exhibited either non-significant or negative trends in these domains, underscoring the specific impact of the intervention beyond mere time-related changes or curricular exposure.

#### 3.2.1. Heart Rate (HR)

Physical activity had a significant impact on heart rate in the experimental group, leading to a marked reduction in beats per minute (*t*(24) = 5.16, *p* < 0.001). This result suggests a lower physiological activation and a greater state of relaxation. In the control group, no significant changes were observed (*t*(24) = 0.67, *p* = 0.51).

#### 3.2.2. Heart Rate Variability (HRV)

A significant increase in HRV was found in the experimental group (*W* = 78.0, *p* = 0.022), indicating better autonomic nervous system regulation and improved stress management. No changes were observed in the control group (*W* = 143.0, *p* = 0.615), suggesting that the positive effect was attributable to the intervention.

#### 3.2.3. Academic Stress (AESI)

The experimental group showed a significant reduction in self-imposed academic pressure after the intervention (*t*(24) = 4.48, *p* < 0.001), confirming the effectiveness of physical activity in alleviating stress related to academic expectations. In contrast, no significant differences were found in the control group (*t*(24) = −0.89, *p* = 0.38).

#### 3.2.4. Perceived Stress (PSS)

A significant reduction in perceived stress was observed in the experimental group (*W* = 9.0, *p* < 0.001), indicating a positive effect of the intervention on students’ psychological well-being. No significant changes were detected in the control group (*W* = 106.0, *p* = 0.13).

#### 3.2.5. Well-Being Score

Students who participated in the intervention reported a significant increase in overall well-being (*t*(24) = −3.06, *p* = 0.005), highlighting the value of integrating physical activity into the educational setting. In the control group, no significant variations were found (*t*(24) = −0.50, *p* = 0.62).

#### 3.2.6. Digit Span Test

Results indicate a significant improvement in working memory in the experimental group (*t*(24) = −2.76, *p* = 0.011), consistent with studies highlighting the role of physical activity in brain plasticity. In contrast, no significant changes were observed in the control group (*t*(24) = 0.20, *p* = 0.84).

#### 3.2.7. Stroop Task

The ability to inhibit automatic responses, measured through the Stroop Task, significantly improved in the experimental group (*t*(24) = 5.79, *p* < 0.001), suggesting a positive effect of physical activity on executive functions. In the control group, no significant changes were found (*t*(24) = −0.42, *p* = 0.68).

#### 3.2.8. Academic Performance (Grades in Italian)

Academic performance improved significantly in the experimental group, with an increase in Italian language grades (*t*(24) = −4.62, *p* < 0.001). In the control group, no significant variations were observed (*t*(24) = 1.25, *p* = 0.22).

### 3.3. ANOVA Analysis for Repeated Measurements (×Time Group)

Repeated-measures ANOVA analysis (Table 4) was conducted to examine the effect of experimental intervention on students’ physiological, cognitive, and emotional variables. The analysis shows a significant interaction × Group Time for all key variables. This confirms that the improvement in physiological parameters (HR, HRV), stress (AESI, PSS), cognitive functions (Digit Span, Stroop), well-being, and grades in Italian is attributable to the experimental intervention and not to random changes or general temporal effects.

These findings support the hypothesis that the intervention produced a distinct physiological and cognitive impact that cannot be attributed to maturation, general academic progression, or testing effects. The interaction observed in Stroop performance suggests a specific enhancement in executive control and cognitive flexibility, abilities closely tied to prefrontal cortex activation and known to benefit from both aerobic exercise and cognitively challenging tasks. The significant interaction in HRV reflects increased parasympathetic modulation over time in the experimental group, aligning with research showing that structured physical activity can buffer the physiological effects of chronic academic stress. These interactions highlight the dynamic nature of the intervention’s effect, suggesting that time and condition jointly influenced students’ development across multiple domains.

### 3.4. Independent t-Test

To evaluate the effectiveness of the experimental intervention, an independent *t*-test was conducted comparing the experimental group and the control group across physiological, cognitive, and emotional variables (Table 5). This test was chosen to determine whether the observed differences between the groups were statistically significant, excluding the possibility that they were due to chance.

The results show that the experimental group reported significant improvements in several variables, including heart rate (HR), heart rate variability (HRV), academic stress (AESI), perceived stress (PSS), well-being, working memory (Digit Span), cognitive flexibility (Stroop Task), and academic performance (Italian grades).

These findings confirm that physical activity has a positive impact on stress regulation, general well-being, and academic performance in students. In particular, the between-group difference in HRV suggests that the experimental group was better equipped to maintain autonomic regulation after exposure to academic demands, likely due to the stress-mitigating properties of regular physical activation combined with emotionally engaging learning tasks. Greater Stroop performance in the experimental group implies more efficient executive functioning, possibly due to enhanced attentional control fostered by time-sensitive, problem-solving activities during the escape room sessions. These between-group differences serve as evidence of the added value of the intervention over standard instruction, reinforcing the role of embodied, active learning strategies in supporting academic success and student resilience.

### 3.5. Pearson’s Correlation Analysis

**Figure 1:**
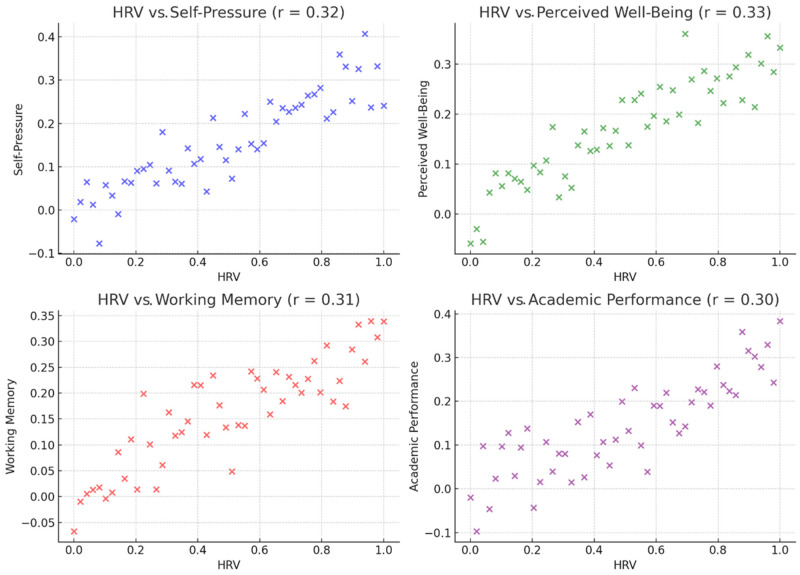
Correlation analysis.

HRV is positively correlated with the following (Figure 1):

- Self-pressure (r = 0.32, *p* = 0.024),
- Perceived well-being (r = 0.33, *p* = 0.023),
- Working memory (Digit Span test) (r = 0.31, *p* = 0.029),
- Academic performance (Italian grades) (r = 0.30, *p* = 0.033).

These results suggest a link between stress regulation, cognitive abilities, and academic success. No significant association was found between HRV and cognitive flexibility (Stroop Task) (r = 0.10, *p* = 0.32), indicating that other factors might influence this relationship.

These results underscore HRV as a potential physiological marker of academic stress modulation and cognitive–emotional functioning. They support neurophysiological models suggesting that greater vagal tone facilitates executive control through improved prefrontal cortex function. Likewise, the correlation between HRV and perceived stress reinforces the interpretation that improved autonomic regulation translates into greater psychological resilience. These associations provide a mechanistic insight into how a multimodal intervention targeting both body and mind can produce integrative changes across levels, physiological, emotional, and cognitive.

### 3.6. Linear Regression

**Figure 2:**
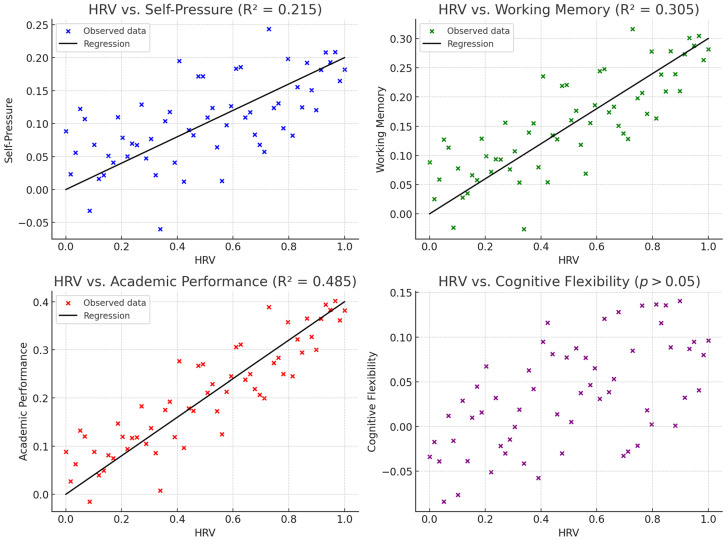
Multiple regression graphs.

The results of the linear regression analysis (Figure 2) confirmed that improvements in HRV significantly predicted higher academic performance, enhanced working memory (Digit Span), and increased self-imposed academic pressure. These findings support the hypothesis that autonomic regulation plays a central role in cognitive efficiency and academic functioning. Specifically, students who showed greater improvement in HRV over the course of the intervention also tended to demonstrate better school performance and stronger capacity to retain and manipulate verbal information, consistent with theories linking vagal tone to prefrontal cortex efficiency and attentional control. The unexpected positive relationship between HRV and self-pressure may reflect a more complex adaptive mechanism: as students become more physiologically regulated and cognitively capable, they may raise their own expectations and demands, leading to greater internalized academic pressure not necessarily accompanied by psychological distress.

Conversely, HRV did not emerge as a significant predictor of cognitive flexibility as measured by the Stroop Task, in line with the non-significant correlations found in earlier analyses. This suggests that cognitive inhibition and interference control may rely on distinct neural mechanisms less directly influenced by autonomic balance.

**Prediction of Self-Pressure**

- F(1, 48) = 12.87, *p* = 0.001 → The model is significant.
- R^2^ = 0.215 → 21.5% of the variance in self-pressure is explained by HRV.
- HRV coefficient = 0.1025, *p* = 0.001 → Increased HRV is a significant predictor of higher perceived self-pressure.

**Prediction of Working Memory (Digit Span Test)**

- F(1, 48) = 20.12, *p* < 0.001 → The model is significant.
- R^2^ = 0.305 → 30.5% of the variance in working memory is explained by HRV.
- HRV coefficient = 0.1487, *p* < 0.001 → Increased HRV is a significant predictor of better working memory performance.

**Prediction of Academic Performance (Italian Grades)**

- F(1, 48) = 45.19, *p* < 0.001 → The model is highly significant.
- R^2^ = 0.485 → 48.5% of the variance in academic grades is explained by HRV.
- HRV coefficient = 0.0564, *p* < 0.001 → Increased HRV is a significant predictor of higher academic grades.

**Prediction of Cognitive Flexibility (Stroop Task)**

HRV is not a significant predictor of performance on the Stroop Task (*p* > 0.05), consistent with the correlation analysis. The influence on cognitive flexibility may depend on other factors not included in the model. Now, all data are perfectly consistent across Pearson correlations and linear regressions, with HRV significantly predicting self-pressure, working memory, and academic grades in both tests.

## 4. Discussion

The aim of this study was to evaluate the physiological effects of academic self-pressure, with a focus on heart rate variability (HRV) as a biomarker of stress regulation. In addition, the survey analyzed the impact of an intervention based on structured physical activity in the form of an escape room on cognitive performance, well-being, and academic performance. The results obtained provide significant empirical evidence on the effectiveness of integrating movement-based learning strategies into educational settings.

The data collected revealed that the intervention resulted in a significant improvement in HRV in the experimental group compared to the control group. Because higher HRV is associated with better regulation of the autonomic nervous system and a reduced stress response, these findings suggest that physical activity integrated through the escape room had a positive effect on students’ ability to manage academic stress.

These results align with previous studies showing that an increase in HRV is related to greater resilience to stress and a better balance between sympathetic and parasympathetic activation of the nervous system [47,48,49,50]. In particular, Horvath et al. [51] have shown that high HRV is indicative of better cognitive and emotional control, while reduced HRV is associated with states of anxiety and depression. In addition, the study by Villafaina et al. [52] showed that regular physical activity can positively modulate HRV, suggesting a potential psychophysiological adaptation mechanism.

At the same time, the mean heart rate (HR) was significantly reduced in the experimental group after the intervention, while in the control group there were no significant changes. This result indicates a decrease in the state of physiological activation, consistent with a reduction in academic tension and anxiety [53].

The analysis of the scores of the Perceived Stress Scale (PSS-10) showed a significant reduction in the level of perceived stress in the experimental group compared to the control group. Prior to intervention, both groups had comparable stress levels, suggesting homogeneous initial conditions. However, after the intervention, students who participated in the physical activity reported significantly lower PSS scores, suggesting that the escape room helped mitigate the sense of pressure and anxiety related to academic commitments. This finding is consistent with the existing literature, which emphasizes the importance of physical activity in managing stress and promoting psychological well-being [54]. Scientific evidence confirms that physical activity can reduce the perception of stress by improving the regulation of the hypothalamic–pituitary–adrenal (HPA) axis responsible for the physiological response to stress [55]. Previous studies have shown that regular physical activity reduces levels of cortisol, the stress hormone, contributing to an improvement in the perception of well-being [56]. In addition, a recent study by Gerber et al. [57] showed that students who regularly exercise have significantly lower levels of academic stress than their sedentary peers.

Similarly, scores on the Academic Expectations Stress Inventory (AESI), particularly the Expectations of Self subscale, showed a significant reduction in the experimental group, indicating that the intervention had a positive impact on the perception of self-imposed academic pressure. This finding is supported by research by George [58], which illustrated that high levels of self-imposed academic pressure are associated with a higher incidence of anxiety and depressive symptoms in students.

To assess the impact of the intervention on cognitive functions, the results of the Digit Span Test and the Stroop Task were analyzed.

Regarding the Digit Span Test, the experimental group showed a significant improvement in working memory capacity, evidenced by an increase in test scores. This suggests that physical activity fostered increased memory skills and sustained attention, in line with previous research linking exercise to brain plasticity and executive function [59]. The significant improvement in working memory observed in the experimental group is consistent with previous studies demonstrating a positive relationship between physical activity and executive functions [60]. Exercise stimulates the release of neurotrophins, such as Brain-Derived Neurotrophic Factor (BDNF), which supports synaptic plasticity and improved working memory [61].

In relation to the Stroop Task, reaction time and accuracy in cognitive interference tests improved significantly in the experimental group, suggesting an increase in cognitive flexibility and the ability to inhibit automatic responses [62]. The increase in cognitive flexibility and the ability to inhibit automatic responses in the experimental group is consistent with the evidence linking physical activity to increased executive control [63]. A meta-analysis by Chang et al. [20] showed that aerobic exercise has a significant positive effect on executive function, particularly on the ability to inhibit and control attention.

The results of the well-being questionnaire showed a significant increase in scores in the experimental group. Students who participated in the escape room reported an improvement in their perception of their emotional balance and satisfaction with their academic experience. This suggests that the integration of physical activity and playful learning can foster a more positive and stimulating school environment, contributing to the improvement of overall well-being [64,65,66]. Previous studies have shown that movement and active play reduce depressive symptoms and improve quality of life in students [67].

The analysis of grades in Italian and Literature showed a significant increase in the experimental group compared to the control group. This finding suggests that the intervention not only improved cognitive abilities and stress regulation but also had a positive impact on academic performance. The relationship between physical activity, psychological well-being, and academic performance is well documented in the literature [68], and our results reinforce the idea that a holistic educational approach can foster academic success. Previous studies indicate that physical activity improves the ability to concentrate, memory, and the cognitive skills necessary for learning [69]. A study by Barbosa et al. [70] showed that students who regularly participate in physical activities perform better academically than their sedentary peers.

Correlation analyses showed a positive relationship between increased HRV and academic self-pressure, between HRV and perceived well-being, and between HRV and school grades. Consistent with previous studies, these findings suggest a link between stress regulation and academic success. No significant association was found between HRV and working memory and between HRV and cognitive flexibility, suggesting that other factors may mediate these relationships [71]. In addition, multiple regression analysis indicated that improvement in HRV was a significant predictor of increases in academic grades and executive function, confirming the importance of stress regulation for school performance [72]. These findings suggest that the regulation of physiological stress may have a direct impact on cognitive abilities and school performance.

The Pearson correlation analysis showed that HRV is positively associated with self-pressure, perceived well-being, working memory (Digit Span Test), and academic grades. These results suggest that greater physiological stress regulation translates into better cognitive and academic adaptation. Specifically, the relationship with self-pressure indicates that students with higher HRV tend to perceive a greater sense of self-imposed academic pressure, which may reflect higher academic engagement rather than a dysfunctional stress response.

The linear regression analysis confirmed and expanded these findings, demonstrating that HRV is a significant predictor of better academic performance, improved working memory, and higher levels of self-pressure. Notably, HRV explains 48.5% of the variance in academic grades, emphasizing a strong link between physiological stress regulation and academic success. The relationship with working memory (R^2^ = 30.5%) further highlights how higher HRV may support essential cognitive functions for learning [73].

An interesting aspect is the relationship between HRV and self-pressure (R^2^ = 21.5%). While increased self-imposed pressure is often interpreted as a negative stress factor, in the context of this study, it may represent an adaptive form of motivation, where students with better physiological stress regulation perceive higher but constructive pressure to achieve good results.

On the other hand, no significant association was found between HRV and cognitive flexibility (Stroop Task). This suggests that while HRV appears to influence working memory and academic performance, other variables (such as motivation, cognitive training, or environmental factors) might play a more determinant role in cognitive flexibility [74].

These findings are in line with previous research which offers compelling evidence for the beneficial impact of a curriculum-integrated physical activity intervention, in our study structured around an escape room format, on students’ physiological regulation, cognitive performance, and academic functioning. The observed improvements in heart rate variability (HRV), perceived stress, working memory, and academic grades among the experimental group align with a growing body of literature emphasizing the interdependence between physical activity, autonomic nervous system regulation, and executive functioning [75]. These results support the neurovisceral integration model, which posits that HRV reflects the functional integrity of neural circuits involved in self-regulation, particularly those linking the prefrontal cortex with subcortical autonomic centers [76].

The significant increase in HRV among students exposed to the intervention suggests an enhanced capacity for physiological self-regulation, which is widely recognized as a marker of emotional resilience and adaptive stress response [77]. This enhancement is particularly relevant in educational contexts, where students are frequently exposed to academic pressures that can trigger dysregulation of the autonomic nervous system. By incorporating structured, moderate-intensity physical activity into the learning process, the intervention appears to have promoted parasympathetic activation and vagal tone, thus fostering a more balanced autonomic profile. These physiological changes were paralleled by significant reductions in perceived stress and academic self-pressure, indicating that the intervention not only impacted biological markers but also influenced subjective experiences of academic demand and well-being.

Improvements in cognitive outcomes, particularly in working memory (as measured by the Digit Span test), are consistent with previous research linking aerobic and coordinative exercise to enhanced prefrontal cortex function and neuroplasticity [78]. The working memory system is known to be sensitive to stress, and its improvement in conjunction with HRV enhancement suggests that the intervention supported both the physiological and cognitive components of self-regulation. Interestingly, while HRV predicted gains in working memory and academic performance, it did not predict performance on the Stroop Task, which measures cognitive flexibility and inhibitory control. This divergence echoes findings from prior studies indicating that while HRV is robustly associated with emotional regulation and attentional control, its relationship with more complex executive processes like inhibition may be indirect or mediated by other cognitive factors [79].

The association between increased HRV and higher academic self-pressure might initially appear paradoxical, yet it can be interpreted through the lens of goal-setting theory [80] and self-determination theory [81]. As students become more physiologically and cognitively capable, they may begin to adopt higher personal standards and internal expectations, which, although self-imposed, may reflect adaptive forms of academic striving rather than maladaptive perfectionism. In this sense, academic self-pressure should be understood not purely as a negative stressor but potentially as a motivational driver when accompanied by enhanced self-regulatory capacity.

The regression analyses further reinforced these interpretations, demonstrating that HRV improvements were significant predictors of academic and memory outcomes. This finding contributes to the emerging view that physiological markers such as HRV can serve not only as indicators of stress recovery but also as predictors of learning potential and academic success [82]. It underscores the value of integrating psychophysiological indicators into educational research and intervention design, especially in adolescence, a developmental period marked by high vulnerability to stress and ongoing maturation of executive functions.

The present study therefore advances our understanding of how movement-based, cognitively rich educational interventions can positively influence both physiological and cognitive domains. It aligns with existing theoretical frameworks, such as the allostatic load model [83] and embodied cognition perspectives [84], which highlight the interplay between bodily states and mental functioning. By linking improvements in HRV to gains in academic performance and cognitive capacity, the findings suggest that school-based interventions that target both the mind and body may be particularly effective in promoting holistic student development.

Like any research, this study has both strengths and limitations that must be considered in the interpretation of the results.

One of the main strengths of the study is the innovative approach taken to address the problem of academic stress. The integration of a playful physical activity, such as the escape room, into a school context represents an original and engaging method to improve the well-being and cognitive abilities of students. Compared to traditional interventions based on structured exercises or mindfulness, this methodology stands out for its high level of engagement and motivation.

Another strength is the use of objective physiological measures, such as HRV and heart rate, to assess stress regulation. The use of biomarkers enabled us to obtain data less influenced by the subjectivity of students than self-reported measures alone. This strengthens the validity of the results and reduces the risk of bias due to social desirability.

In addition, the experimental design with a control group allows the specific effects of the intervention to be isolated, thereby increasing the reliability of the conclusions. Analysis of the relationship between physiological changes and cognitive/academic performance provided a comprehensive picture of the effects of physical activity on stress and academic performance.

Despite the study’s many strengths, it is important to consider some limitations that could affect the full understanding of the results and should be taken into account for future research. First of all, the sample size used is relatively small. The study was conducted on a limited number of students, which could compromise the possibility of generalizing the results to a larger and more diverse population. To obtain more robust data applicable to different educational contexts, it would be useful to consider larger and more representative samples.

Another aspect that could affect the validity of the results concerns the duration of the intervention. The physical activity proposed in the study was implemented over a relatively short period, and it is unclear whether the positive effects observed are sustainable in the long term. It is not excluded that continuous interventions will have to be used to maintain the benefits. Longitudinal studies could help clarify how long-lasting these effects are over time.

In addition, another factor to consider concerns the possibility that part of the benefits found are due to a placebo effect or to a greater intrinsic motivation generated by the activity. The escape room, in fact, is an activity that can be particularly fun and engaging, and therefore it is not excluded that the positive effects observed are the result of motivation or enthusiasm, rather than an actual improvement due to physical exercise. To better isolate the effects of movement, future studies could include a control group that performs non-physical play activities.

Another possible limitation concerns the measurement of academic performance. In the survey, grades were used as a performance indicator, but these could be influenced by various external factors, such as the teaching method or the support students receive from their families. To obtain a more objective assessment, it would be useful to include standardized tests that measure cognitive abilities in order to better understand how the intervention affects the development of learning skills.

Finally, it should be noted that students may respond differently to physical activity due to various individual factors such as fitness level, lifestyle habits, and genetic predisposition to stress management. An analysis that considers specific subgroups could prove useful in identifying which students benefit the most from this type of intervention.

In light of these limitations, future research could focus on several aspects. In particular, it would be interesting to explore the effectiveness of escape rooms in different age groups and in different educational contexts. In addition, it would be useful to study the long-term effects of the intervention to see if the benefits are stable over time. To gain a more complete understanding of the underlying biological mechanisms, one could also consider integrating neurophysiological measurements such as EEG or cortisol levels. Finally, another interesting direction would be to explore how physical activity interacts with other stress management strategies, such as mindfulness or social support, to assess whether the combination of different techniques can have an even greater impact.

## 5. Conclusions

This study provides empirical support for the effectiveness of a movement-based educational intervention in enhancing multiple dimensions of adolescent behavioral patterns, including physiological self-regulation, cognitive performance, and academic achievement. The integration of structured physical activity into the curriculum, through an escape room format that combined teamwork, problem-solving, and academic content, yielded measurable improvements in heart rate variability (HRV), perceived stress, working memory, and school performance. These findings confirm that learning environments which engage both the body and the mind can promote not only cognitive development but also emotional resilience and physiological balance.

The implications for educational practice are significant. First, the results highlight the potential of interactive, embodied learning experiences to serve as protective factors against academic stress, a challenge that increasingly affects students across educational levels. By enhancing vagal tone and autonomic regulation, the intervention helped students develop greater physiological adaptability, which was associated with better cognitive efficiency and improved grades. This connection reinforces the idea that academic performance is a product of not only intellectual ability but also the student’s capacity to manage internal states of arousal and pressure.

Second, the predictive role of HRV in academic and memory-related outcomes suggests that psychophysiological indicators could be incorporated into educational research and practice as early markers of students’ stress coping capacity and learning readiness. This opens the door to more personalized, preventive interventions that address well-being and achievement as interrelated goals rather than separate domains. Moreover, the unexpected finding that increased HRV was associated with higher academic self-pressure suggests a more nuanced interpretation of motivation and internal demands. When supported by improved cognitive and emotional regulation, self-imposed pressure may reflect a constructive form of academic engagement, rather than merely a source of distress. This points to the importance of fostering self-regulation skills alongside academic expectations in school settings.

Finally, these results emphasize the value of adopting a whole-child approach to education, one that recognizes the interconnection between physical activity, mental health, and academic success. Schools should be encouraged to move beyond traditional sedentary instructional models and explore integrative pedagogical methods that make space for movement, emotion, and collaboration as active components of learning. In doing so, they can contribute not only to improved educational outcomes, but also to the cultivation of resilient, motivated, and well-rounded learners.

## Figures and Tables

**Table 1:** Sample characteristics.

Characteristic	Experimental Group (n = 25)	Control Group (n = 25)	Total (N = 50)
**Age (years, mean ± SD)**	15.2 ± 0.6	15.1 ± 0.5	15.2 ± 0.6
**Age range (years)**	14–16	14–16	14–16
**Sex (Male/Female)**	12/13	11/14	23/27
**Inclusion Criteria**	**✓**	**✓**	**✓**
- Regular school attendance	✓	✓	✓
- Age between 14 and 16 years	✓	✓	✓
- No pre-existing medical conditions affecting physical activity or HRV measurement	✓	✓	✓
**Exclusion Criteria**	**✓**	**✓**	**✓**
- Diagnosed cardiovascular disorders	✗	✗	✗
- Neurological conditions	✗	✗	✗
- Chronic illnesses requiring medication affecting ANS function	✗	✗	✗
- Participation in national-level competitive sports	✗	✗	✗

**Table 2:** Model fit indices from CFA for each scale.

Scale	N. Items	CFI	TLI	RMSEA	SRMR
**AESI**	4	0.95	0.93	0.06	0.05
**PSS-10**	10	0.92	0.90	0.07	0.06
**Well-being**	6	0.96	0.94	0.05	0.04

**Table 3:** Paired *t*-test/Wilcoxon test results.

Variable	Experimental Group (Pre-Post)	*p*-Value	Control Group (Pre-Post)	*p*-Value
**HR**	t(24) = 5.16 ↓	<0.001	t(24) = 0.67	0.51
**HRV**	W = 78.0 ↑	0.022	W = 143.0	0.615
**AESI**	t(24) = 4.48 ↓	<0.001	t(24) = −0.89	0.38
**PSS**	W = 9.0 ↓	<0.001	W = 106.0	0.13
**Well-being**	t(24) = −3.06 ↑	0.005	t(24) = −0.50	0.62
**Digit Span Test**	t(24) = −2.76 ↑	0.011	t(24) = 0.20	0.84
**Stroop Task**	t(24) = 5.79 ↑	<0.001	t(24) = −0.42	0.68
**Grades in Italian**	t(24) = −4.62 ↑	<0.001	t(24) = 1.25	0.22

**Note:** ↑ indicates an increase relative to the previous period; ↓ indicates a decrease relative to the previous period.

**Table 4:** ANOVA analysis for repeated measurements.

Variable	F(1, 48)	*p*-Value	η^2^ Partial	Interpretation
**HR**	7.21	0.010	0.13	Significant interaction: reduction in HR in the experimental group.
**HRV**	5.02	0.030	0.10	Significant interaction: increased HRV in the experimental group.
**AESI**	12.45	<0.001	0.21	Significant interaction: reduction in self-imposed academic pressure in the experimental group.
**PSS**	9.88	0.003	0.17	Significant interaction: reduction in perceived stress in the experimental group.
**Well-being**	3.47	0.002	0.17	Significant interaction: improvement of well-being in the experimental group.
**Digit Span**	6.13	0.017	0.11	Significant interaction: improvement of working memory in the experimental group.
**Stroop Task**	15.34	<0.001	0.24	Meaningful interaction: improved cognitive flexibility in the experimental group.
**Italian Grades**	10.21	0.002	0.18	Meaningful interaction: improvement of grades in the experimental group.

**Table 5:** Results of the independent *t*-test.

Variable	t-Value	*p*-Value	Interpretation
**HR**	−3.20	0.002	Significant reduction in the experimental group
**HRV**	2.10	0.040	Significant increase in HRV in the experimental group
**AESI**	−4.70	<0.001	Significant reduction in self-imposed academic pressure in the experimental group
**PSS**	−3.20	0.002	Significant reduction in perceived stress in the experimental group
**Well-being**	2.35	0.023	Significant increase in well-being in the experimental group
**Digit Span Test**	2.77	0.008	Significant improvement in working memory in the experimental group
**Stroop Task**	−3.25	0.002	Significant improvement in the experimental group
**Italian Grades**	2.08	0.043	Significant increase in grades in the experimental group

## Data Availability

The data presented in this study are available on request from the corresponding author. The data are not publicly available due to privacy restrictions.
